# Upregulated functional gene expression programmes in tumour pericytes mark progression in patients with low‐grade glioma

**DOI:** 10.1002/1878-0261.13016

**Published:** 2021-06-05

**Authors:** Clara Oudenaarden, Jonas Sjölund, Kristian Pietras

**Affiliations:** ^1^ Division of Translational Cancer Research Department of Laboratory Medicine Lund University Cancer Centre Lund University Sweden

**Keywords:** computational biology, glioblastoma, pericytes, perivascular niche, tumour microenvironment

## Abstract

Pericytes conceivably play important roles in the tumour microenvironment of glioblastoma multiforme (GBM) by allowing for an aberrant vasculature and acting as a component in the perivascular niche that supports glioma stem‐like cells. However, a lack of specific markers has hampered in‐depth elucidation of the functional contribution of pericytes to GBM. This study provides a comprehensive computational biology approach to annotate pericyte marker genes in the GBM vasculature through integration of data from single‐cell RNA‐sequencing studies of both mouse and human tissue, as well as bulk tumour and healthy tissue gene expression data from patients with GBM. We identified distinct vascular‐ and immune‐related gene expression programmes in tumour pericytes that we assessed for association with GBM characteristics and patient survival. Most compellingly, pericyte gene signatures that were upregulated in tumours compared with normal brain tissue were indicative of progression of low‐grade gliomas into high‐grade glioma, suggested by a markedly shorter overall survival. Our results underline the functional importance of tumour pericytes in low‐grade glioma and may serve as a starting point for efforts for precision targeting of pericytes.

## Graphical abstract

Here, we provide a comprehensive computational annotation of brain vascular pericytes in the glioblastoma multiforme (GBM) tumour microenvironment. We revealed distinct vascular‐ and immune‐related gene expression programmes in tumour pericytes by integrating single‐cell RNA‐sequencing data of both mouse and human tissues, and tumour and healthy tissue gene expression data from patients with GBM. Moreover, high expression of upregulated gene signatures in tumour pericytes was enriched in isocitrate dehydrogenase wild‐type and O6‐methylguanine‐DNA methyltransferase unmethylated tumours, was associated with a shorter overall survival and was indicative of the progression from low‐grade to high‐grade GBM.

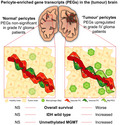

**Abbreviations**
ANPEP: alanine‐aminopeptidase
BBB: blood–brain barrier
CSPG4: chondroitin sulphate proteoglycan 4
GBM: glioblastoma multiforme
GEPIA2: gene expression profiling interactive analysis 2
GO: gene ontology
IDH: isocitrate dehydrogenase
LGG: low‐grade glioma
MGMT: O6‐methylguanine‐DNA methyltransferase
NG2: neural/glial antigen 2
NVU: neurovascular unit
OPC: oligodendrocyte precursors
PDGFRβ: platelet‐derived growth factor beta
PEG: pericyte‐enriched gene transcript
PVN: perivascular niche
scRNA‐seq: single‐cell RNA sequencing
TCGA: the cancer genome atlas
UMAP: uniform manifold approximation and projection for dimension reduction
VEGF: vascular endothelial growth factor

## 1 Introduction

Gliomas are brain tumours that arise from glial cells or their precursors, such as astrocytes (astrocytomas), oligodendrocytes (oligodendrogliomas), and ependymal cells (ependymomas) [1, 2]. Notably, the highly vascularized glioblastoma multiforme (GBM), classified by the WHO as a grade IV astrocytoma, displays a poor prognosis, with an average life expectancy of 14.6 months after diagnosis despite the best available treatment efforts [3, 4]. The destructive nature of GBM can partly be explained by an aberrant and dysfunctional vasculature as a result of high levels of tumour‐secreted vascular endothelial growth factor (VEGF), contributing to tumour aggressiveness in multiple ways [5, 6]. Firstly, a notably disrupted blood–brain barrier (BBB) with fewer tight junctions and a reduced vessel coverage of pericytes lead to vessel permeability and leakiness. Insufficient delivery of oxygen and nutrients, as well as increasing intratumoral pressure, is known to drive tumour invasiveness [6, 7]. Secondly, glioblastoma stem‐like cells reside in the perivascular niche (PVN), where their self‐renewing potential is maintained by the proximity to the vasculature [8]. Moreover, glioblastoma stem‐like cells have been shown to be a robust producer of VEGF themselves, thereby further enhancing the disorganized and leaky vasculature [9]. Since therapy‐resistant glioblastoma stem‐like cells are a major determinant of the recurrence of GBM after initial treatment, research has been focused on the endothelial cells in order to disrupt the PVN. However, direct targeting of the endothelium with the anti‐angiogenic neutralizing VEGF antibody bevacizumab has not proven to be the promised solution so far [10].

Besides the endothelium, the pericyte constitutes another pivotal cellular component of the PVN. Pericytes are elongated, spindle‐shaped cells that are embedded within the vascular basement membrane and circumscribe the endothelial cell layer of capillaries and pre‐ and postcapillary structures [11]. In contrast to the flattened morphology and perpendicular orientation of vascular smooth muscle cells, pericytes have a round cell body and lay along the longitudinal axis of the vessels, thus providing support and stability [12, 13]. Especially in the brain, vessel integrity is of high importance, and with a ratio of one pericyte for every one to three endothelial cells, the brain vasculature is one of the most mural cell‐saturated tissues of the human body [11]. The high density of pericytes strongly contributes to the support and maintenance of the BBB [14, 15, 16]. Notably, the BBB tightly regulates the homeostasis of the central nervous system through the neurovascular unit (NVU), composed of endothelial cells, pericytes, and astrocytic end‐feet [17]. The central position of the pericytes in the NVU allows for efficient communication between pericytes and the other constituent cell types. In addition, brain pericytes have shown to be capable of responding to neuronal signals, such as neurotransmitters, and having contractile properties, which enables regulation of the stiffness and diameter of the vessel wall per capillary segment, thereby influencing the local blood supply when necessary [18, 19]. Despite histological evidence for their key position in the brain vasculature, pericyte marker genes and proteins are still conspicuously missing, hampering functional studies.

In the tumour context, pericytes are often loosely associated with the endothelium and are less abundant when compared to normal blood vessels, aggravating the leaky phenotype of the tumour microvasculature and contributing to the hypoxic and infiltrative nature of the tumour [20, 21]. Studies on the role of pericytes in the glioma microenvironment have contributed to a divergent spectrum of functions. On the one hand, fewer pericytes and a disrupted BBB may support glioma progression by dysregulation of nutrient transport, providing a rationale for improving the pericyte coverage as a therapeutic strategy. On the other hand, a high pericyte density conceivably offers a physical barrier, preventing drugs from reaching their intended target. Hence, ablation of GBM‐derived pericytes would benefit intratumoral delivery of chemotherapeutic agents due to increased vessel permeability [22]. Moreover, an elevated pericyte vessel coverage has been observed in response to anti‐angiogenic treatment, subsequently counteracting the therapeutic efficacy by supplying the endothelial cells with survival factors, suggesting pericytes as possible additional targets for improved anti‐angiogenic treatment [23, 24].

In this study, we aimed to shed light on previously unknown role(s) of pericytes in GBM and explored the prognostic relevance of GBM pericyte signatures in low‐grade gliomas (LGGs; grades II and III) as early indicators for tumour progression. By defining pericyte‐enriched gene transcripts (PEGs) using single‐cell RNA‐sequencing (scRNA‐seq) data and extracting correlated transcripts from the cancer genome atlas (TCGA)/PanCancer Atlas GBM data set, we analysed shared gene expression patterns among tumour pericytes and made functional comparisons with gene expression programmes in normal brain pericytes. In addition to the expected vascular gene programmes expressed by pericytes, tumour pericytes were also associated with gene programmes related to tumour immune functions. The tumour pericyte signatures, but not the normal pericyte signatures, were strongly represented among grade III glioma patients that harboured genomic alterations prognostic of tumour progression and drug resistance, such as status on isocitrate dehydrogenase (IDH) and O6‐methylguanine‐DNA methyltransferase (MGMT). Indeed, functional gene signatures from tumour pericytes were correlated with a worse overall survival in grade III glioma patients when compared to the normal pericyte signatures. These results underline the functional importance of tumour pericytes in LGG to grade IV GBM progression and suggest their influence on patient survival.

## 2 Methods

### 2.1 Selection of gene transcripts enriched in pericytes

Gene transcripts enriched in brain pericytes were selected from a single‐cell RNA‐seq data set on murine brain that is accessible at http://betsholtzlab.org/VascularSingleCells/database.html [25, 26]. Gene expression in pericytes was compared with the combined expression of other cell types present in the brain (astrocytes, oligodendrocytes, microglia, endothelial cells, smooth muscle cells, fibroblast‐like cells), with a gene expression threshold in pericytes of > 100 average counts. The top 200 genes with the highest fold change were selected.

### 2.2 Selection of human PEGs with single‐cell RNA‐sequencing analysis

Each of the top 200 differentially expressed genes in murine brain pericytes was further examined using an adapted analysis pipeline of the online‐accessible visualization of a human GBM single‐cell RNA‐seq data set (http://www.gbmseq.org/) [27]. This data set encompasses astrocytes, oligodendrocytes, neurons, oligodendrocyte precursors (OPCs), neoplastic cells, myeloid cells, and vascular cells. From the 200 mouse genes, only genes that demonstrated the highest gene expression in the human vascular cells were included for downstream analyses. The vascular cells were further divided into pericytes and endothelial cells through additional computational analysis on the same human GBM single‐cell data set (Fig. S1). The single‐cell RNA‐sequencing profiling of human GBM [27] was downloaded from the GEO database (GEO: GSE84465). Cells were filtered based on the cell‐type annotation of the original GBM data set. The data sets were then normalized and scaled using the basic pipeline of seurat (version 4.0.0, [28]). The identified clusters were visualized on a 2D map produced with the uniform manifold approximation and projection for dimension reduction (UMAP) method. Vascular cells were further subdivided into pericytes and endothelial cells based on the expression of well‐defined marker genes. For the analysis of the expression pattern of the 62 genes in human GBM cell types, the average expression of all cells within a cluster was calculated and visualized in a heatmap.

### 2.3 Categorization of differentially expressed human pericyte genes

The expression levels of the differentially expressed human pericyte genes were analysed in their expression in glioblastoma patients (*n* = 163) and normal brain tissue (*n* = 207) from the TCGA and genotype‐tissue expression projects, respectively, through the web‐based tool Gene Expression Profiling Interactive Analysis 2 (GEPIA2; http://gepia2.cancer‐pku.cn/) [29]. Individual genes were annotated as upregulated, downregulated or nonsignificant in GBM patients as compared to normal brain tissue (*P* < 0.01).

### 2.4 Extraction of correlated gene lists

Each of the 62 genes differentially expressed in human brain pericytes was analysed through the cBioPortal for Cancer Genomics (http://cbioportal.org) [30]. The study selected for genomic analysis was GBM (TCGA, PanCancer Atlas) containing 592 samples (585 patients, of which 160 patients have information on mRNA expression). Positively correlated genes to each of the 62 genes that were enriched in human brain pericytes were extracted. Top 200 ranked genes with the highest positive Spearman correlation of each of the genes were used for downstream analyses. This corresponded to *P*‐values ranging between 0.004 and 1.43 × 10^−13^ for the 200th gene of each of the 62 correlated gene lists. See Table S1 for a full list of *P*‐values for each of the 200th correlated gene.

### 2.5 Generation of heatmaps with hierarchical clustering

Overlap between correlated genes of every possible gene pair combination among the 62 selected genes ((62 × 62)/2 = 3844 combinations) was analysed with the use of http://bioinformatics.psb.ugent.be/webtools/Venn/. Results were separated between the previously annotated categories ‘upregulated’ and ‘nonsignificant’ gene expression in GBM patients and documented in Excel. Subsequently, the data were uploaded to the freely available web server heatmapper.ca [31]. Heatmaps were constructed with the following settings: scale type ‘none’, clustering method ‘complete linkage’ and ‘average linkage’, distance measurement method ‘Euclidian’, apply clustering to ‘rows and columns’.

### 2.6 Expression heatmap of 62 PEGs across different brain cell types

Mouse whole brain and hippocampus SMART‐seq data (gene expression aggregated per cluster, calculated as trimmed means) from the Allen Brain Atlas consortium were downloaded on 14 October 2020 (https://portal.brain‐map.org/atlases‐and‐data/rnaseq/mouse‐whole‐cortex‐and‐hippocampus‐smart‐seq) [32, 33]. Genes of interest were selected and compiled into a heatmap with Excel. For expression of Pvalb and Sst neurons, the average was calculated of 13 and 40 cell clusters, respectively.

### 2.7 Spatial RNA‐seq data processing, visualization and integration

A human GBM spatial transcriptomic (Visium v1 chemistry) data set was obtained from 10X genomics (https://support.10xgenomics.com/spatial‐gene‐expression/datasets/1.2.0/Parent_Visium_Human_Glioblastoma) and processed according to a standardized pipeline provided by Seurat (https://satijalab.org/seurat/articles/spatial_vignette.html) in r [34]. In short, data were normalized using SCTransform and principal component analysis and UMAP dimensionality reduction (using dimensions 1–30) were all done using default parameters.

The PEG signatures were quantified as the average *Z*‐score of member genes and visualized using Seurat's SpatialFeaturePlot function. For the integration of the Darmanis *et al*. scRNA‐seq GBM data set with the spatial transcriptomic data set, we preprocessed the scRNA‐seq data as a reference and performed label transfer as outlined in the Seurat protocol. For each of the scRNA‐seq‐derived groups, in this case pericytes, the procedure generates a probabilistic classification for each position, that is a prediction score. The pericyte‐annotated clusters from the scRNA‐seq data set could thus be projected onto the spatial tissue image using SpatialFeaturePlot function as above.

### 2.8 Gene ontology analysis

Gene ontology (GO) analyses were performed with Metascape on *Homo sapiens* according to the settings of ‘Express Analysis’, as well as with Enrichr [35, 36, 37]. Analysis was performed on the overlapping correlating genes within each determined PEG signature (Table S2).

### 2.9 Immune cell signature analysis

Cellular expression of gene lists was investigated among eleven hematopoietic cell types with the addition of several types of stromal cells with the online data browser provided by the Immunological Genome Project (ImmGen) [38].

### 2.10 Analysis of chromosomal alterations and survival

Level 3 LGG and GBM RNA‐seq data were obtained from the TCGA consortium (https://portal.gdc.cancer.gov/). RNA‐seq data processing was performed in Rstudio version 1.1.463. Secondary or recurrent GBM and LGG samples were excluded from the analysis. The expression of each gene signature was considered low for patients with expression values in the 1st quartile and high for patients in the 4th quartile. The log‐rank test was used for Kaplan–Meier overall survival analyses, and *P*‐values of < 0.05 were considered to denote statistically significant differences. MGMT methylation status was obtained from Verhaak and colleagues [39]. Chi‐squared analysis with Yates' continuity correction as appropriate was used to assess genotype distributions between the groups.

## 3 Results

### 3.1 Annotation of pericyte‐enriched gene transcripts from glioma patients

**Fig. 1:**
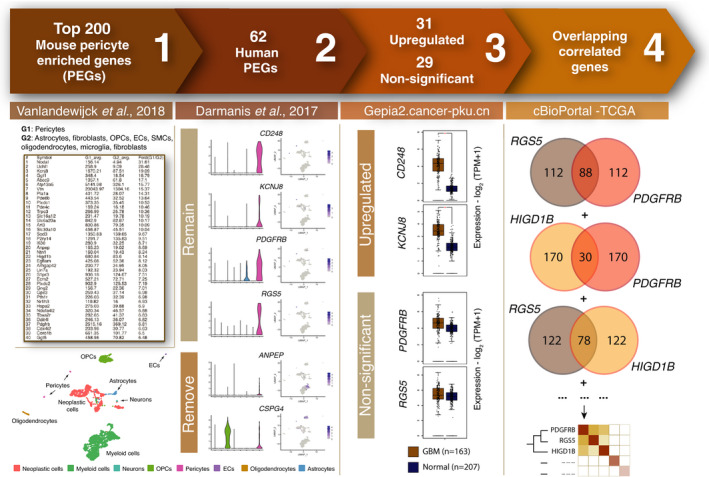
Visual representation of the workflow. (1) The top 200 genes highly expressed in mouse brain pericytes were extracted from http://betsholtzlab.org/VascularSingleCells/database.html [25, 26]. (2) Cell type expression of genes from step 1 was examined in human GBM patients [27]. Only genes that displayed highest expression among vascular cells (orange bars) remained for further analyses. (3) Genes were categorized as upregulated or nonsignificant based on their gene expression levels in GBM tissue (brown) as compared to normal brain tissue (blue; *P* < 0.01) [29]. (4) Correlated gene lists to each of the 62 previously selected genes were extracted from cBioPortal utilizing the TCGA GBM data set. The number of overlapping correlated genes between every existing gene pair was visualized in a heatmap with hierarchical clustering.

Since our aim was to focus specifically on functional gene expression programmes manifested in brain tumour pericytes, the first challenge was to define a set of pericyte core gene transcripts as a starting point for further exploration. Identification of pericytes has traditionally required combined analysis of their morphology, location and marker expression. Commonly used pericyte markers include platelet‐derived growth factor receptor beta (PDGFRβ), neural/glial antigen 2 [NG2/chondroitin sulphate proteoglycan 4 (CSPG4)], alanine‐aminopeptidase (ANPEP/CD13), alpha smooth muscle actin (αSMA/ACTA2), and desmin [40]. However, none of these markers are fully inclusive of, or exclusive to, pericytes, generating challenges for a representative detection of pericytes. In order to include more putative pericyte gene transcripts, we created a workflow that combined data of several scRNA‐seq studies, as well as the TCGA data on GBM patients (Fig. 1).

First, we analysed a single‐cell RNA transcriptomic data set generated from murine brain containing 1088 validated pericytes [25, 26]. We extracted a list of genes that were substantially higher expressed in pericytes, as compared to other cell types in the brain, including smooth muscle cells, microglia, fibroblasts, oligodendrocytes, and endothelial cells. We selected the top 200 PEGs with the largest difference in expression compared with the average expression of all other cell types and with an expression threshold of > 100 average counts to avoid artefactual outliers (Fig. 1.1; Table S1).

**Fig. 2:**
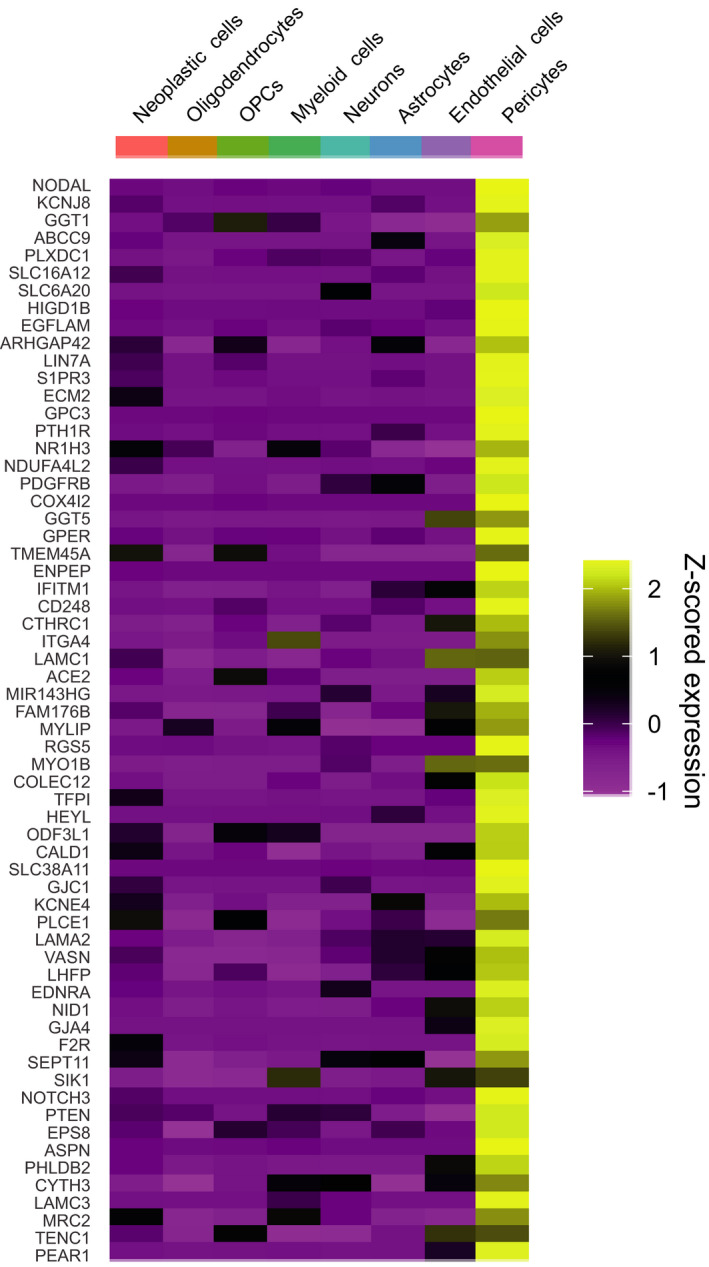
Expression of mouse PEGs in human (tumour) brain cell types. Heatmap displaying 62 mouse PEGs that have their highest expression in the pericyte cluster of our adapted analysis of the data set from Darmanis *et al*. [27].

Subsequently, we ensured that the mouse gene list corresponded to genes that displayed an enriched expression in human brain tumour pericytes. To this end, we analysed which of the 200 mouse PEGs were expressed mainly by pericytes in an scRNA‐seq analysis of four GBM patients by Darmanis *et al*. [27]. Firstly, we annotated each cell included in the three vascular clusters from the original study with higher resolution, thus defining one of the clusters as endothelial cells and two as pericytes (Fig. S1A–C). From the 200 mouse PEGs, only genes of which the highest expression level was detected in the two pericyte clusters were further considered as human PEGs (Figs 1.2 and 2), resulting in 62 human PEGs that remained for further analyses (genes marked in bold in Table S1). Commonly used pericyte markers such as *PDGFRB* and *CD248* remained in our final list of human PEGs. Others, such as *ANPEP* and *CSPG4*, also showed a high expression among other constituent cell types of the brain, such as myeloid cells and OPCs, respectively, and were therefore removed from the curated list (Fig. 1.2). The majority of these PEGs were also expressed predominantly by pericytes in the Allen mouse brain atlas transcriptomic data sets (Fig. S2) [33, 41].

As a third step in our approach, we enriched for PEGs with functional relevance in GBM by investigating the individual gene expression of the 62 human PEGs in GBM tissue as compared to normal brain tissue and categorized the genes as either statistically ‘upregulated’, ‘downregulated’ or ‘nonsignificant’ (using a threshold of *P* < 0.01; Fig. 1.3) [29]. Out of the 62 PEGs, 31 genes were designated as upregulated, including *CD248* and *KCNJ8*, and 29 genes as nonsignificant, including the established pericyte markers *PDGFRB*, *ABCC9* and *RGS5*. Two genes were found to be downregulated and were not considered further (*SLC38A11* and *PTHR1*; Table S1).

**Fig. 3:**
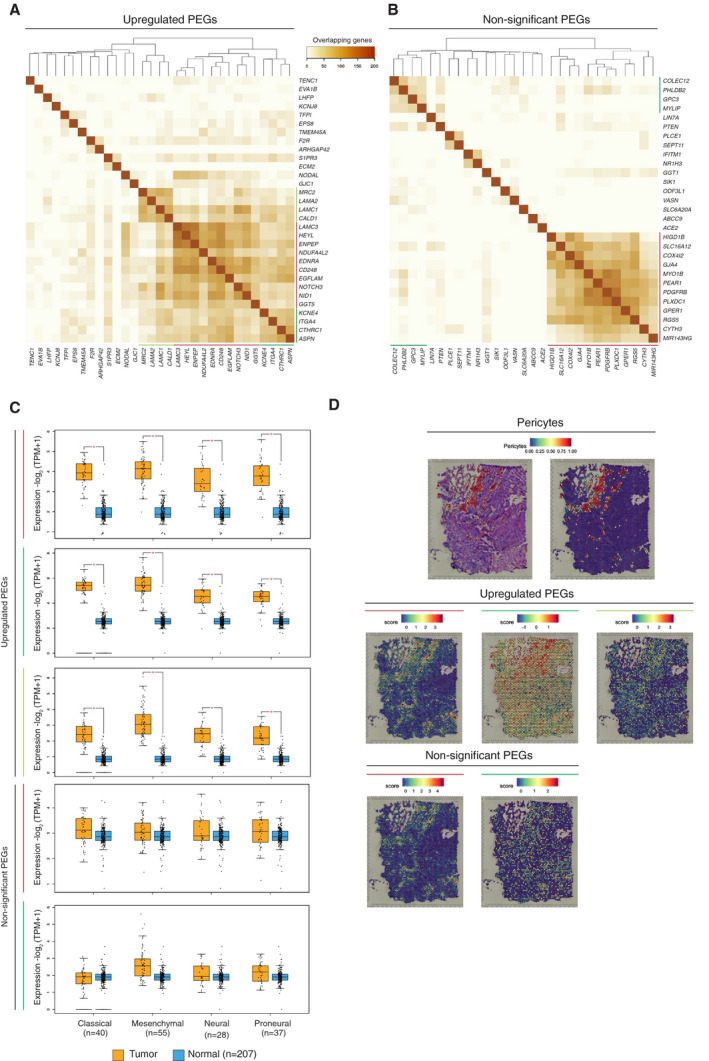
Identification of clusters among PEGs. The number of overlapping correlated genes of upregulated PEGs (A) and nonsignificant PEGs (B) is visualized as heatmaps with hierarchical clustering according to the method of complete linkage. Coloured lines indicate functionally distinct PEG clusters. Data were visualized using heatmapper.ca [31]. (C) Expression levels of each of the five PEG signatures for the classified GBM subtypes. Coloured lines match to the coloured lines from panels A and B. Statistical significance (*P* < 0.01) was determined by one‐way analysis of variance (ANOVA) and is indicated with an asterisk. Data were visualized with GEPIA2 [29]. (D) Spatial RNA‐sequencing visualization of the Darmanis‐pericyte signature and the five PEG signatures from panels A and B, with matching coloured lines.

Finally, to gain functional insight for each of the PEGs, we extracted a list of the most highly correlated transcripts from the TCGA PanCancer Atlas GBM data set containing 592 samples (including 160 samples with mRNA expression) through cBioPortal [30]. From each list of correlated genes, we selected the top 200 genes with the highest positive Spearman correlation value. Subsequently, we analysed the number of overlapping correlated genes between each combination of two PEGs and illustrated the relationship between PEGs in a heatmap based on the number of common correlated genes (Fig. 1.4). With the intention to uncover units of functional PEGs, complete linkage hierarchical clustering was performed (Fig. 3A,B). Other clustering methods, such as average linkage, resulted in similar clustering (Fig. S3A,B).

### 3.2 Pericyte‐enriched gene transcripts form distinct functional programmes

The clustering of PEGs exhibited three distinct collections of genes for the upregulated PEGs and two separate clusters for the nonsignificant PEGs (Fig. 3, coloured lines). Accordingly, the genes that constituted each cluster had a large degree of overlap in their correlating genes, indicating that each group signified a functional unit, hereafter also referred to as a signature. All PEG signatures were equally represented across the established GBM subtypes (classical, mesenchymal, neural, proneural), and the upregulated PEG signatures were confirmed to have a higher expression in the GBM samples as compared to the corresponding normal brain tissue in all molecular subtypes (Fig. 3C) [29]. To ensure that the PEG signatures truly represented transcripts that were enriched by pericytes, we overlaid the expression of the five PEG signatures on a human GBM spatial transcriptomic data set (https://support.10xgenomics.com/spatial‐gene‐expression/datasets/1.2.0/Parent_Visium_Human_Glioblastoma), using a gene signature from the pericyte cluster of the data set by Darmanis *et al*. as a reference. Indeed, the expression of each of the five PEG signatures was enriched in the areas identified to contain pericytes, thus validating our approach (Fig. 3D).

### 3.3 Upregulated and nonsignificant ‘vascular’ PEG signatures

**Fig. 4:**
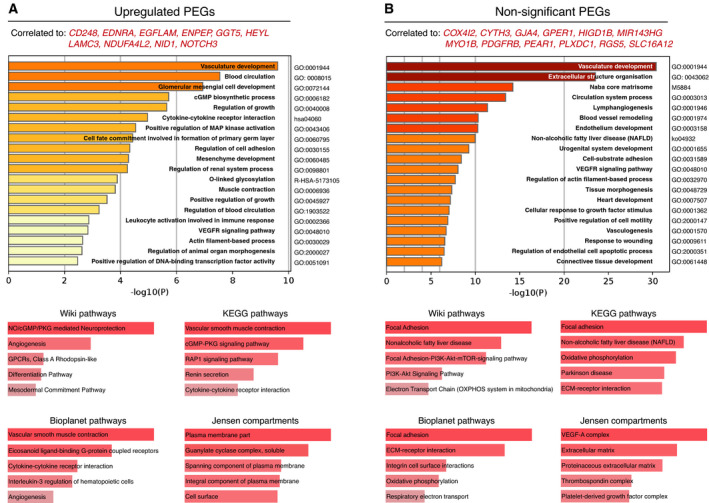
Vascular clusters of PEGs. GO term analysis of vascular upregulated PEGs (A) and vascular nonsignificant PEGs (B). Data were visualized using Metascape (top) and Enrichr (bottom). Coloured bars represent *P*‐values with increasing statistical significance with an increasing bar‐length.

Next, we explored in more detail the functional commonality of the overlapping genes (Table S2) with GO term analysis through Metascape and performed additional pathway analyses with Enrichr [35, 36, 37]. First, we explored the GO terms of the major clusters present among the upregulated and nonsignificant PEGs (Fig. 3A,B, red lines). Not surprisingly, the most prominent upregulated PEG cluster (*CD248*, *EDNRA*, EGFLAM, *ENPEP*, *GGT5*, *HEYL*, *LAMC3*, *NDUFA4L2*, *NID1*, *NOTCH3*) was associated with the vasculature, associating with biological processes in the pathway analyses such as ‘vasculature development’, ‘angiogenesis’, and ‘vascular smooth muscle contraction’ (Fig. 4A). Similarly, the largest association between the nonsignificant PEGs also composed a vascular cluster (*COX4I2*, *CYTH3*, *GJA4*, *GPER1*, *HIGD1B*, *MIR143HG*, *MYO1B*, *PDGFRB*, *PEAR1*, *PLXDC1*, *RGS5*, *SLC16A12*), with the joint conclusion of the pathway analyses demonstrating typical vascular‐related processes, showing main functional annotations such as ‘focal adhesion’, ‘vasculature development’, and ‘extracellular structure organization’ (Fig. 4B).

### 3.4 Two specific upregulated PEG signatures related to the immune system

**Fig. 5:**
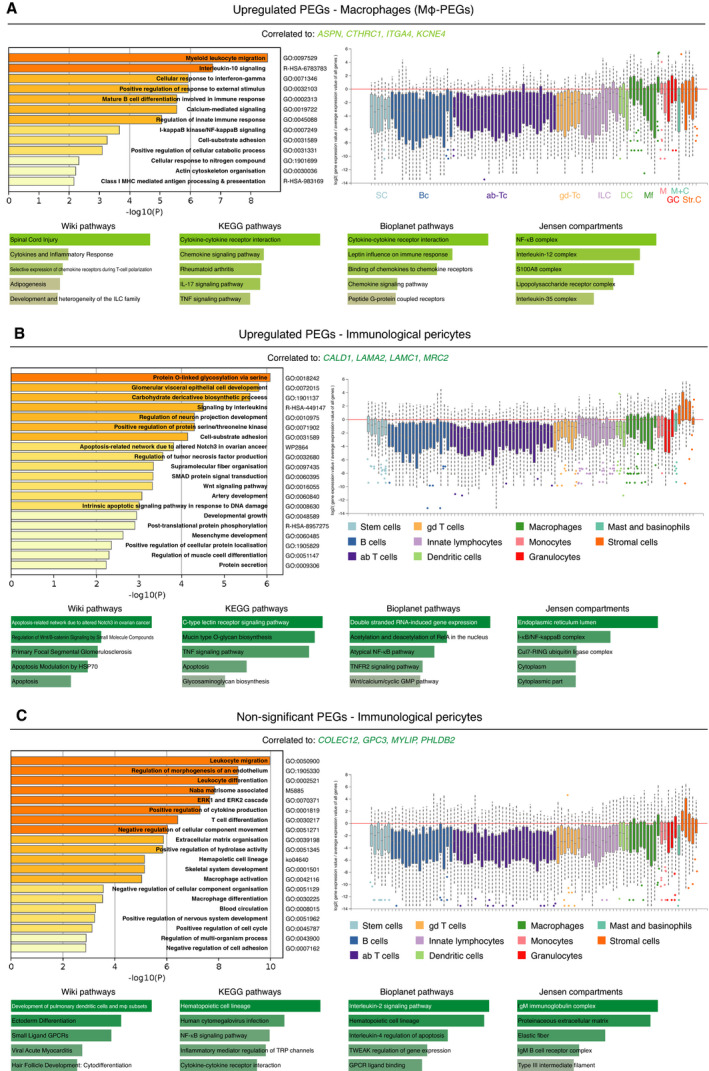
Immune clusters of PEGs. (A–D) GO term analysis with Metascape and Enrichr on common correlated genes to upregulated PEGs (A, B) and nonsignificant PEGs (C). Shades of green correspond to colour‐coding of the individual immune signature. Waterfall plot displays eleven hematopoietic cell types and their gene expression level of common correlated genes. SC, stem cells (light blue). Bc, B cells (blue). Ab‐Tc, alpha‐beta T cells (purple). Gd‐Tc, gamma‐delta T cells (yellow). ILC, innate lymphocytes (light purple). DC, dendritic cells (light green). Mf, macrophages (green). M, monocytes (pink). GC, granulocytes (red). M + C, mast and basophils (cyan). Str. C, stromal cells (orange). Data were visualized using Metascape, Enrichr, and ImmGen [31, 35, 38].

In addition to the vascular gene clusters, we identified clusters of PEGs constituted of shared correlated genes that were strongly associated with immune processes (Fig. 3A,B, green lines, and Fig. 5A–C). Specifically, there were two immune‐associated gene clusters formed by the upregulated PEGs (dark green: *ASPN*, *CTHRC1*, *ITGA4*, *KCNE4,* and light green: *CALD1, LAMA2, LAMC1, MRC2*) and one by the nonsignificant PEGs (dark green: *COLEC12, GPC3, MYLIP, PHLDB2*). In order to make functional distinctions between these immune signatures, we utilized cell expression analysis on a range of hematopoietic cells with ImmGen, in addition to the prior Metascape analysis and gene set enrichment analysis by Enrichr (Fig. 5) [35, 36, 37, 38]. Besides the expected enrichment of the genes correlated with the light green immune upregulated PEG signature to stromal cells, there was an evident association with macrophage populations (waterfall plot in Fig. 5A). In addition, the Metascape enrichment analysis resulted in macrophage‐related terms, such as ‘myeloid leukocyte migration’. The association with macrophages was further supported by the biological pathway analysis, which included IL10, IL17, NF‐κB, and TNF‐α signalling (Fig. 5A). IL17C is known to be produced by macrophages and to subsequently attract monocytes and lead to the transcription of inflammatory genes, such as IFNγ TNF‐α, and IL6 [42, 43, 44]. In addition, the anti‐inflammatory interleukin IL10, also known as cytokine synthesis inhibitory factor, is produced by macrophages and is a key inhibitory signal to inflammatory responses [45]. For this reason, we decided to name this light green upregulated PEG signature the macrophage‐PEG (mφ‐PEG).

Our finding of functional gene programmes in tumour pericytes related to immune responses may indicate previously unappreciated roles for pericytes in immune regulation. However, although the PEGs were selected due to their predominant expression in pericytes, the functional gene programmes derived from the common correlating genes also included genes that were expressed by other constituent cell types within the tumour microenvironment, such as myeloid cells. Conceivably, a pericyte‐derived gene product may thus act to recruit a specific immune cell type, resulting in a high correlation between that PEG signature and genes expressed by the particular immune cell, which could be the case for the upregulated mφ‐PEG signature. On the contrary, we observed a second immune‐associated upregulated PEG signature (Fig 3A, dark green line) of which the correlated genes were almost exclusively expressed by stromal cells (Fig. 5B), while still being associated with immunological processes, indicating that the immune functions may be executed by the pericytes themselves. Interestingly, enriched processes within these immune‐regulatory pericytes include signalling by the NF‐κB, Wnt, and TNF signalling pathways. Similarly, a signature consisting of nonsignificant PEGs (Fig. 3B, dark green line) could also be classified as immunological pericytes, since high expression levels in pericytes were accompanied by immunological processes such as leukocyte migration and differentiation, and positive regulation of cytokine production (Fig. 5C).

Taken together, our functional gene network analysis related PEGs to functions in both the vasculature and in the immune system.

### 3.5 High expression of the upregulated PEG signatures corresponds with worse survival in grade III glioma patients

**Fig. 6:**
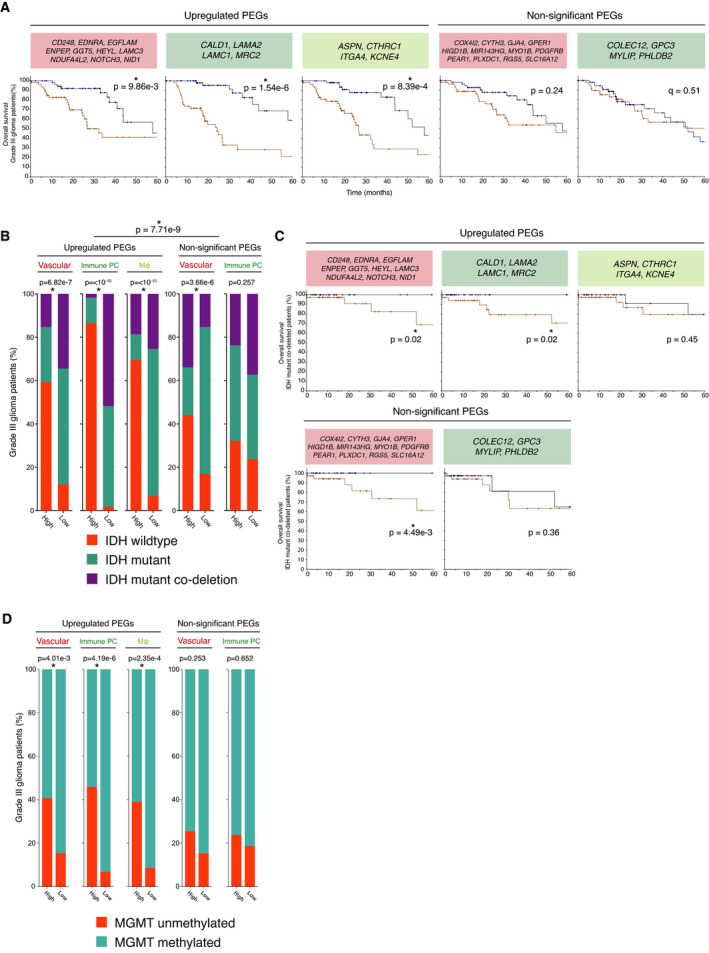
Association between expression of PEGs with a worse prognosis. (A) Kaplan–Meier analysis of overall survival (log‐rank test *P*‐value < 0.05) among the highest (brown) and lowest (blue) quartile of grade III patients (*n* = 59 per arm). (B) IDH status of grade III glioma patients that are high or low expressing (high: *n* = 59; low: *n* = 59) for upregulated and nonsignificant PEGs. Significant chi‐squared test *P*‐values (< 0.05) are indicated with an asterisk. (C) Kaplan–Meier analysis of overall survival (log‐rank test *P*‐value < 0.05) among the highest (brown) and lowest (blue) quartile of IDH mutant codeleted patients (*n* = 42 per arm). (D) MGMT promoter methylation status of grade III glioma patients. Data were visualized in r using the TCGA/PanCancer Atlas data set ‘Brain lower grade glioma’, and statistical significance was determined with Pearson's chi‐squared test (*P* < 0.05).

Next, we set out to elucidate whether the pericyte signatures harboured prognostic value for glioma outcome. In order to investigate the effect of the PEG signatures on glioma progression, we analysed an LGG data set and a GBM data set. We separated the patients of the LGG data set into grade II and grade III patients, so we could analyse overall survival over a broad spectrum of glioma stages. Strikingly, a higher expression of any of the upregulated PEG signatures correlated with significantly shorter overall survival in grade III patients, but not in grade IV GBM or grade II glioma patients, indicating a possible functional role for pericytes during the progression from an LGG to a high‐grade glioma (Fig. 6A and Fig. S4A). In sharp contrast, the expression of the nonsignificant PEG signatures did not exhibit any relation to outcome in neither LGG nor GBM (Fig. 6A and Fig. S4A).

### 3.6 High expression of upregulated PEG signatures correlates with IDH wild‐type patients and MGMT methylation status

In addition to the traditional grading system, the WHO recently designated the genetic mutation status of IDH as clinically relevant for assessing the prognosis of brain tumour patients [4, 46]. With the additional codeletion of chromosomal arms 1p and 19q, IDH‐mutated gliomas are associated with the best prognosis, whereas IDH wild‐type tumours indicate the worst survival outcome. Interestingly, our analysis revealed that high expression of the upregulated PEG signatures was significantly enriched in grade III glioma patients with the worst prognosis, that is, IDH wild‐type, when compared to the nonsignificant PEG signatures (*P* = 7.71e‐9; Fig. 6B), an association that was not present among grade II glioma patients (Fig. S4B). In a similar fashion, typical chromosomal aberrations in GBM patients, such as loss of chromosome 10 and 9p, and gain of chromosome 7, were profoundly represented among grade III glioma patients with high expression of any of the upregulated PEG clusters (Fig. S5A). In striking contrast, the nonsignificant PEG signatures were not related to a particular genetic lesion to the same extent, indicating that pericyte function, rather than mere abundance, was related to the genetic status of the tumour (Fig. S5B).

To investigate the additional relevance of the PEG signatures on overall survival to the initial IDH classification, we performed an additional survival analysis and separated 1066 patients from a merged LGG and GBM TCGA data set based on IDH status. It was evident that all patients with the status of IDH wild‐type had a poor prognosis, independent of PEG signature (Fig. S6A). However, low expression of the upregulated vascular and immunological PEG signatures adds a survival advantage to patients with IDH mutant codeleted status (Fig. 6C).

Another important biomarker for GBM is the methylation status of the MGMT promoter, where unmethylated MGMT is associated with a worse prognosis due to development of drug resistance [47, 48]. High expression of any of the three upregulated PEG signatures in grade III glioma patients was associated with unmethylated MGMT (Fig. 6D) and to a lesser extent in the grade IV GBM and grade II glioma patients (Fig. S6B), supporting our data that upregulated PEG signatures are associated with a worse prognosis in grade III glioma patients.

Taken together, our analyses indicate a strong relationship between functional upregulated gene expression programmes in pericytes and established outcome predictors in low‐grade brain tumours.

## 4 Discussion

In light of the known heterogeneity of pericyte marker expression and morphology, it is not surprising that we found different functional gene programmes among glioma pericytes. Conceivably, the different functions ascribed here to tumour pericytes through diverse transcriptional programmes could either represent different cellular states of pericytes or indicate the existence of pericyte subpopulations. To further resolve these possibilities, employment of scRNA‐seq or high‐resolution multiplexed spatial tracing of indicative marker mRNAs or proteins will be increasingly valuable. However, pericytes appear to be underrepresented in current scRNA‐seq data sets from various malignancies, underscoring the need for the development of specific protocols to release pericytes into single‐cell suspensions and to preserve their viability.

The prototypical function of pericytes is their relevance to blood vessel stability and their established molecular interactions with endothelial cells [11]. Accordingly, we observed clearly defined gene expression signatures related to vascular function(s) both among upregulated and nonsignificant PEGs. Apart from PEG signatures related to the vasculature, we identified pronounced immune signatures among the commonly correlated genes for PEGs. The association between pericytes and immune cells has been investigated to a lesser extent than its engagement with the vasculature. Nevertheless, it is known that pericytes aid in leukocyte trans‐endothelial migration and even exhibit intrinsic phagocytic abilities [49, 50, 51]. Intriguingly, correlated immune genes of upregulated PEGs suggested an immune‐suppressive action, rather than the immune‐supportive function that pericytes are known to exert in physiological situations. In addition, one immune signature related to upregulated PEGs was indicative of macrophage signalling. Macrophages have been intimately linked with GBM progression and invasion, suggesting that pericytes may be close accomplices in this endeavour [52, 53, 54]. Our observations add to previous studies indicating that the activated state of pericytes in GBM is associated with an increased production of anti‐inflammatory cytokines, such as IL‐10 and TGF‐β [55]. Thus, exploration of pericyte targeting in conjunction with immunotherapeutic approaches for GBM is warranted. Finally, our selection strategy for PEGs used normal brain tissue as a reference to define marker genes for pericytes. Conceivably, there may be additional transcripts that are absent from pericytes in normal tissues, but abundantly expressed by tumour pericytes. Thus, efforts to transcriptionally profile pericytes from both GBM and normal brain tissue should be further pursued.

The most compelling finding of our study of enriched pericyte genes in glioma is the clinical relevance of our deduced signatures for LGG patients. We uncovered that high expression of the upregulated PEG signatures was associated with poor survival, especially among grade III glioma patients. Conceivably, this suggests a tumour‐promoting gene expression programme in pericytes, where the activation of certain signalling pathways, for example the upregulated PEG signatures, facilitates glioma growth and local infiltration as a result of the interaction with the tumour microenvironment. Moreover, the genotypic manifestation of upregulated PEGs in grade III patients could represent an early state of progression of grade III lesions into GBM. This hypothesis is further supported by the presence of typical GBM genomic alterations (gain of Chr7, loss of Chr10 and 9p), as well as IDH wild‐type and unmethylated MGMT status associated with the upregulated PEG signatures. Another clinically relevant observation regarding the PEG signatures is the better chance of overall survival for low expression of either the vascular or immunological pericyte signature among IDH mutant codeleted patients. Analysis of these upregulated PEG profiles warrants further investigation to determine whether these signatures could represent an independent prognostic tool for intervention besides the current standard of care of grade III glioma patients, potentially preventing progression of higher‐risk LGGs into high‐grade gliomas and GBM.

Collectively, our comprehensive analysis of pericyte‐enriched genes in human glioma points out that pericytes harbour a heterogenic array of functional gene expression programmes that may indicate substantial cellular heterogeneity. The annotation of specific gene lists of tumour PEGs represents a first advance towards a more complete understanding of pericyte heterogeneity and spatial localization. The genetic and functional differences between upregulated and nonsignificant PEG programmes further demonstrate that generalization of pericytes as therapeutic targets could lead to undesired results, and future focus should lie in developing drugs that would suppress upregulated PEG signatures indicative of poor survival in gliomas. Our study may thus serve as a starting point for further functional analyses of pericyte subsets in order to better understand how to exploit pericyte features for the benefit of glioma patients.

## 5 Conclusion

In this study, we identified that vascular‐ and immune‐associated gene programmes upregulated in pericytes are functionally linked to relevant processes in tumour development, and that they were specifically associated with glioma progression. Hereby, we provide a strong rationale for the development of precision targeting of pericytes in order to counteract the upregulation of pericyte gene programmes that evidently contribute to tumour progression.

## Conflict of interest

The authors declare no conflict of interest.

## Author contributions

CO and KP were responsible for conception of the study. All the authors took part in data analysis, interpretation, and writing or revising of the manuscript.

### Peer Review

The peer review history for this article is available at https://publons.com/publon/10.1002/1878‐0261.13016.

## Supporting information


**Fig. S1**. Subdivision of vascular clusters in a transcriptomic GBM dataset. (A) Feature plot of scRNA‐seq analysis on 4 GBM patients [27]. Vascular clusters (orange) can be divided between endothelial (ECs) and pericyte (PCs) clusters (arrows). (B and C) Expression of five prototypical pericyte genes (B) and five prototypical endothelial cell genes (C) are visualized on the feature plot of panel A and matching violin plots below.

**Fig. S2**. Pericyte enriched gene transcripts in a murine transcriptomic dataset of the Allen Brain Atlas. Heatmap displaying 62 mouse PEGs that have their highest expression in the pericyte cluster of SMART‐seq dataset on mouse whole brain from the Allen Brain Atlas.

**Fig. S3**. Visualisation of clusters of PEGs with average linkage. (A, B) The number of overlapping correlated genes of upregulated PEGs (A) and non‐significant PEGs (B) is visualized as heatmaps with hierarchical clustering according to the method of average linkage. Coloured lines are corresponding to functionally distinct PEG clusters based on complete linkage displayed in Fig. 2. Data was visualized using heatmapper.ca [30].

**Fig. S4**. Overall survival and IDH status of PEG signatures in grade II and IV glioma patients. (A) Percentage of overall survival (logrank test *P*‐value < 0.05) among the highest (brown) and lowest (blue) quartile of grade II glioma and grade IV GBM patients (grade II glioma: *n* = 53 each arm; grade IV GBM: *n* = 38 each arm). (B) IDH status of grade II glioma patients that are high or low expressing (*n* = 53 each arm) for upregulated and non‐significant PEGs. Statistical significance *P*‐values (< 0.05) were determined with the chi‐squared test and are indicated with an asterisk.

**Fig. S5**. Characteristic high‐grade glioma genetic aberrations for the PEG signatures in grade III glioma patients. (A, B) Percentage of high and low expressing grade III glioma patients of upregulated (A) non‐significant PEGs (B) is displayed for each of the PEG signatures. Significant chi‐squared test *P*‐values (< 0.05) are indicated with an asterisk. Data was visualized using cBioPortal.

**Fig. S6**. Overall survival and MGMT status of PEG signatures in glioma patients. (A) Percentage of overall survival (logrank test *P*‐value < 0.05) among merged cohort of the highest (brown) or lowest (blue) quartile of glioma patients (IDH wildtype *n* = 57 each arm; IDH mutant *n* = 63 each arm). Dataset in cBioPortal: Merged Cohort of LGG and GBM, TCGA, Cell 2016. (B) MGMT promoter methylation status of grade II glioma and grade IV GBM patients. Data was visualized in R using the TCGA/PanCancer Atlas datasets ‘Brain Lower Grade Glioma’ and ‘GBM’. Statistical significance was determined with Pearson’s chi‐squared test (*P* < 0.05).

**Table S1**. PEGs.
**Table S2**. Overlapping correlating genes of the PEGs.

## Data Availability

The data that supports the findings of this study are available in the supplementary material of this article.
