# Supplementary material for: Upregulated functional gene expression programmes in tumour pericytes mark progression in patients with low‐grade glioma
Source: Mol Oncol. 2021 Jun 5;16(2):405–21. doi: 10.1002/1878-0261.13016 (PMC8763650; doi:10.1002/1878-0261.13016)
Supplement: Supplementary file 7 — Table S1. PEGs. Table S2. Overlapping correlating genes of the PEGs. [file MOL2-16-405-s004.zip › mol2_13016_Supplementary Table 1 - Pericyte genes.docx]

**Supplementary Table 1. Pericyte-enriched gene transcripts (PEGs)**

a. Top 200 enriched genes in pericytes in comparison with smooth muscle cells, microglia, fibroblast-like cells, oligodendrocytes, endothelial cells, and astrocytes. Cut off expression threshold in pericytes set to 100. Vanlandewijck *et al.*, 2018, Methods Mol Biol.

b. Cell type that showed highest gene expression according to single cell RNA sequencing analysis on human GBM patients Darmanis *et al.*, 2017, Cell Rep.

c. Gene expression comparison between GBM and normal brain tissue. NS: not significant

d. Human equivalent is Slc6a20

e. Mouse long-coding RNA gene, no human equivalent to utilize for downstream analysis

f. GSTT1 was not recognized by GEPIA2. No alias available.

g. KIAA0247 used for highest cell type expression. Susd6 used for comparison between GBM and normal brain tissue on GEPIA2 analysis.

| Position | Gene | Fold change*^a^* | Highest expression*^b^* | GBM expr.*^c^* | Spearman  p value |  |
| --- | --- | --- | --- | --- | --- | --- |
| **1** | ***Nodal*** | **31.61** | **Pericytes** | **Up** | **3.8417E-06** |  |
| 2 | *Uchl1* | 28.48 | Neurons | Down | - |  |
| **3** | ***Kcnj8*** | **19.09** | **Pericytes** | **Up** | **5.82E-07** |  |
| **4** | ***Ggt1*** | **18.79** | **Pericytes** | **NS** | **0.000428009** |  |
| **5** | ***Abcc9*** | **17.1** | **Pericytes** | **NS** | **2.67E-07** |  |
| 6 | *Atp13a5* | 15.77 | OPCs | NS | - |  |
| 7 | *Vtn* | 15.37 | Astrocytes | Down | Down |  |
| 8 | *Pla1a* | 14.31 | Endothelial cells | NS | 5.08E-08 |  |
| 9 | *Pde8b* | 13.64 | Neurons | NS | - |  |
| **10** | ***Plxdc1*** | **10.53** | **Pericytes** | **NS** | **6.50E-09** |  |
| 11 | *Pde4c* | 10.46 | Neurons | Down | - |  |
| 12 | *Trpc3* | 10.36 | Neurons | NS | - |  |
| **13** | ***Slc16a12*** | **10.19** | **Pericytes** | **NS** | **0.000107733** |  |
| **14** | ***Slc6a20a^d^*** | **10.17** | **Pericytes** | **NS** | **2.76E-07** |  |
| 15 | *Art3* | 10.09 | Oligodendrocytes | NS | - |  |
| 16 | *Slc30a10* | 10.04 | Astrocytes | NS | - |  |
| 17 | *Sod3* | 9.67 | Astrocytes | NS | - |  |
| 18 | *P2ry14* | 9.51 | Astrocytes | NS | - |  |
| 19 | *Ifi30* | 8.71 | Myeloid | Up | - |  |
| 20 | *Anpep* | 8.69 | Myeloid | Up | - |  |
| 21 | *Ntn1* | 8.24 | OPCs | Up | - |  |
| **22** | ***Higd1b*** | **8.14** | **Pericytes** | **NS** | **2.76E-10** |  |
| **23** | ***Egflam*** | **8.12** | **Pericytes** | **Up** | **1.22E-07** |  |
| **24** | ***Arhgap42*** | **8.05** | **Pericytes** | **Up** | **9.13E-09** |  |
| **25** | ***Lin7a*** | **8.03** | **Pericytes** | **NS** | **0.000681921** |  |
| **26** | ***S1pr3*** | **7.51** | **Pericytes** | **Up** | **8.85708E-06** |  |
| **27** | ***Ecm2*** | **7.25** | **Pericytes** | **Up** | **6.74E-12** |  |
| 28 | *Plxdc2* | 7.19 | Myeloid | Up | - |  |
| 29 | *Gng2* | 7.01 | Neurons | NS | - |  |
| **30** | ***Gpc3*** | **6.98** | **Pericytes** | **NS** | **1.66241E-06** |  |
| **31** | ***Pth1r*** | **6.98** | **Pericytes** | **Down** | **4.83356E-05** |  |
| **32** | ***Nr1h3*** | **6.93** | **Pericytes** | **NS** | **2.15E-12** |  |
| 33 | *Hspa2* | 6.90 | Oligodendrocytes | NS | - |  |
| **34** | ***Ndufa4l2*** | **6.88** | **Pericytes** | **Up** | **1.86E-07** |  |
| 35 | *Tbxa2r* | 6.83 | Neurons | Up | - |  |
| 36 | *Ddit4l* | 6.82 | Astrocytes | NS | - |  |
| **37** | ***Pdgfrb*** | **6.81** | **Pericytes** | **NS** | **3.33E-11** |  |
| **38** | ***Cox4i2*** | **6.63** | **Pericytes** | **NS** | **5.45E-08** |  |
| 39 | *Coro1b* | 6.50 | Myeloid | Up | - |  |
| **40** | ***Ggt5*** | **6.48** | **Pericytes** | **Up** | **3.44E-07** |  |
| 41 | *Slc19a1* | 6.47 | Astrocytes | NS | 1.44E-09 |  |
| 42 | *Gpx8* | 6.21 | Endothelial cells | Up | 2.59E-12 |  |
| **43** | ***Gper1*** | **6.18** | **Pericytes** | **NS** | **2.3064E-05** |  |
| **44** | ***Tmem45a*** | **6.10** | **Pericytes** | **Up** | **2.38E-07** |  |
| 45 | *AW549542^e^* | 6.08 | - | - | - |  |
| **46** | ***Enpep*** | **5.96** | **Pericytes** | **Up** | **5.54E-09** |  |
| 47 | *Chn1* | 5.85 | Astrocytes | Down | - |  |
| 48 | *Atp2a3* | 5.72 | Neurons | NS | - |  |
| 49 | *Oaf* | 5.71 | Astrocytes | NS | - |  |
| 50 | *Nbl1* | 5.70 | Neurons | Down | - |  |
| 51 | *Angptl2* | 5.58 | OPCs | Up | - |  |
| **52** | ***Ifitm1*** | **5.57** | **Pericytes** | **NS** | **7.34E-10** |  |
| 53 | *G0s2* | 5.56 | Neurons | NS | - |  |
| 54 | *Ptn* | 5.47 | Astrocytes | Up | - |  |
| 55 | *Sema6d* | 5.40 | Astrocytes | NS | - |  |
| **56** | ***Cd248*** | **5.38** | **Pericytes** | **Up** | **5.41E-12** |  |
| 57 | *Akap7* | 5.36 | Neurons | NS | - |  |
| **58** | ***Cthrc1*** | **5.21** | **Pericytes** | **Up** | **1.20E-08** |  |
| **59** | ***Itga4*** | **5.13** | **Pericytes** | **Up** | **3.47E-11** |  |
| 60 | *Pald1* | 5.12 | Myeloid | Up | - |  |
| 61 | *Grm3* | 5.10 | Astrocytes | Down | - |  |
| 62 | *Gucy1b3* | 5.10 | Neurons | Down | - |  |
| **63** | ***Lamc1*** | **5.02** | **Pericytes** | **Up** | **5.28E-12** |  |
| 64 | *Snx33* | 5.01 | OPCs | Up | - |  |
| 65 | *Aif1l* | 5.01 | Oligodendrocytes | NS | - |  |
| 66 | *Atp1a2* | 5.01 | Astrocytes | Down | - |  |
| **67** | ***Ace2*** | **4.98** | **Pericytes** | **NS** | **0.003508537** |  |
| 68 | *Dtx3* | 4.95 | Neurons | NS | - |  |
| 69 | *Hspa12a* | 4.95 | Neurons | Down | - |  |
| 70 | *Ankrd50* | 4.93 | Neurons | NS | - |  |
| 71 | *Cspg4* | 4.93 | OPCs | Up | - |  |
| 72 | *Csk* | 4.92 | Myeloid | Up | - |  |
| 73 | *Adap2* | 4.90 | Myeloid | Up | - |  |
| 74 | *Pdzd2* | 4.86 | OPCs | Down | - |  |
| 75 | *Rgs4* | 4.76 | Neurons | Down | - |  |
| 76 | *Rarres2* | 4.76 | Neoplastic | Up | - |  |
| 77 | *Klhl23* | 4.75 | Neurons | NS | - |  |
| 78 | *Naalad2* | 4.74 | Neurons | Down | - |  |
| **79** | ***Mir143hg*** | **4.71** | **Pericytes** | **NS** | **1.82E-11** |  |
| 80 | *Pcdh18* | 4.60 | Neurons | Up | - |  |
| 81 | *Slco3a1* | 4.57 | Neurons | NS | - |  |
| 82 | *Rab3a* | 4.56 | Neurons | Down | - |  |
| 83 | *Tmem86a* | 4.56 | Neurons | Up | - |  |
| 84 | *Il34* | 4.56 | Neurons | Down | - |  |
| **85** | ***Eva1b*** | **4.48** | **Pericytes** | **Up** | **7.98E-07** |  |
| **86** | ***Mylip*** | **4.46** | **Pericytes** | **NS** | **9.38E-09** |  |
| 87 | *Vstm4* | 4.38 | Neurons | NS | - |  |
| 88 | *Ldlr* | 4.36 | OPCs | NS | - |  |
| 89 | *Sdc3* | 4.35 | OPCs | Up | - |  |
| 90 | *Gprc5c* | 4.34 | Astrocytes | NS | - |  |
| **91** | ***Rgs5*** | **4.31** | **Pericytes** | **NS** | **5.48E-07** |  |
| 92 | *Limd1* | 4.25 | OPCs | Up | Up |  |
| **93** | ***Myo1b*** | **4.20** | **Pericytes** | **NS** | **1.96E-10** |  |
| **94** | ***Colec12*** | **4.20** | **Pericytes** | **NS** | **1.04E-09** |  |
| **95** | ***Tfpi*** | **4.17** | **Pericytes** | **Up** | **0.000184291** |  |
| **96** | ***Heyl*** | **4.12** | **Pericytes** | **Up** | **7.39E-08** |  |
| 97 | *Pitpnc1* | 4.11 | Astrocytes | NS | - |  |
| 98 | *Gypc* | 4.07 | Myeloid | Up | - |  |
| **99** | ***Odf3l1*** | **4.04** | **Pericytes** | **NS** | **6.11401E-05** |  |
| 100 | *Slc12a2* | 4.01 | Oligodendrocytes | NS | - |  |
| 101 | *Pid1* | 3.99 | OPCs | NS | - |  |
| 102 | *Glrb* | 3.98 | Neurons | NS | - |  |
| 103 | *Fam118b* | 3.94 | Astrocytes | Up | - |  |
| **104** | ***Cald1*** | **3.87** | **Pericytes** | **Up** | **1.10E-08** |  |
| 105 | *Arhgef17* | 3.86 | Neurons | Down | - |  |
| **106** | ***Slc38a11*** | **3.83** | **Pericytes** | **Down** | **4.44152E-06** |  |
| 107 | *Dock6* | 3.82 | Endothelial cells | NS | 1.37E-09 |  |
| 108 | *Tmem134* | 3.81 | Neoplastic cells | NS | - |  |
| **109** | ***Gjc1*** | **3.79** | **Pericytes** | **Up** | **5.13E-08** |  |
| 110 | *Btbd3* | 3.67 | Oligodendrocytes | NS | - |  |
| 111 | *Slc9a3r1* | 3.65 | Astrocytes | NS | - |  |
| 112 | *Tbc1d1* | 3.65 | Myeloid | NS | 9.05E-09 |  |
| 113 | *Daam1* | 3.63 | Neurons | NS | - |  |
| 114 | *Ptpn9* | 3.61 | Astrocytes | Up | - |  |
| 115 | *Dlc1* | 3.61 | Astrocytes | NS | - |  |
| **116** | ***Kcne4*** | **3.61** | **Pericytes** | **Up** | **4.97118E-06** |  |
| 117 | *Efhd2* | 3.51 | Myeloid | NS | - |  |
| **118** | ***Plce1*** | **3.49** | **Pericytes** | **NS** | **6.96E-11** |  |
| **119** | ***Lama2*** | **3.47** | **Pericytes** | **Up** | **3.54E-09** |  |
| **120** | ***Vasn*** | **3.45** | **Pericytes** | **NS** | **1.02E-10** |  |
| 121 | *Sept4* | 3.42 | Oligodendrocytes | Down | - |  |
| 122 | *Itga1* | 3.39 | Endothelial cells | Up | 3.41E-10 |  |
| 123 | *Perp* | 3.39 | Endothelial cells | NS | 5.86E-08 |  |
| 124 | *Cpm* | 3.38 | Oligodendrocytes | Up | 8.79E-11 |  |
| 125 | *Acsl1* | 3.38 | Myeloid | NS | - |  |
| 126 | *Sdc2* | 3.37 | Astrocytes | Up | - |  |
| 127 | *Ctdspl* | 3.35 | Astrocytes | NS | - |  |
| 128 | *Ephx1* | 3.35 | Astrocytes | NS | - |  |
| 129 | *Tnfaip1* | 3.35 | Neurons | NS | - |  |
| 130 | *Sh2b3* | 3.33 | Myeloid | Up | - |  |
| 131 | *Plcl1* | 3.31 | Oligodendrocytes | Down | - |  |
| 132 | *Arhgdib* | 3.30 | Myeloid | Up | - |  |
| **133** | ***Lhfp*** | **3.29** | **Pericytes** | **Up** | **1.43412E-05** |  |
| 134 | *Sept7* | 3.28 | Olidodendrocytes | NS | - |  |
| **135** | ***Ednra*** | **3.27** | **Pericytes** | **Up** | **2.34E-07** |  |
| 136 | *Sh3glb2* | 3.26 | Neurons | Down | - |  |
| **137** | ***Nid1*** | **3.25** | **Pericytes** | **Up** | **3.35E-09** |  |
| 138 | *Inpp4b* | 3.24 | Neurons | NS | - |  |
| **139** | ***Gja4*** | **3.23** | **Pericytes** | **NS** | **1.01E-08** |  |
| 140 | *Ggta1* | 3.22 | Myeloid | NS | - |  |
| **141** | ***F2r*** | **3.21** | **Pericytes** | **Up** | **4.22488E-05** |  |
| 142 | *Imp3* | 3.18 | Neurons | NS | - |  |
| 143 | *Dmd* | 3.16 | Neurons | NS | - |  |
| 144 | *Ptk2* | 3.15 | Oligodendrocytes | NS | - |  |
| 145 | *Ptprk* | 3.15 | Oligodendrocytes | NS | - |  |
| 146 | *Cog7* | 3.14 | OPCs | NS | - |  |
| 147 | *Tnfrsf21* | 3.14 | OPCs | Up | - |  |
| **148** | ***Sept11*** | **3.14** | **Pericytes** | **NS** | **2.99E-08** |  |
| 149 | *Pkd2* | 3.13 | Neurons | NS | - |  |
| 150 | *Kcnmb1* | 3.12 | Myeloid | Up | - |  |
| 151 | *Pcdh19* | 3.11 | OPCs | Down | - |  |
| **152** | ***Sik1*** | **3.10** | **Pericytes** | **NS** | **6.26E-07** |  |
| 153 | *Pld1* | 3.09 | Oligodendrocytes | NS | - |  |
| **154** | ***Notch3*** | **3.08** | **Pericytes** | **Up** | **1.04E-11** |  |
| 155 | *Slc1a5* | 3.08 | Myeloid | Up | - |  |
| **156** | ***Pten*** | **3.05** | **Pericytes** | **NS** | **9.93594E-06** |  |
| 157 | *Lama4* | 3.05 | Endothelial cells | Up | 3.79E-10 |  |
| 158 | *Wls* | 3.04 | Astrocytes | Up | - |  |
| 159 | *Poldip2* | 3.03 | Neurons | NS | - |  |
| 160 | *Ehd3* | 3.02 | OPCs | Down | - |  |
| 161 | *Mfge8* | 3.01 | Astrocytes | NS | - |  |
| 162 | *Notch1* | 2.93 | OPCs | Up | - |  |
| 163 | *Gstt2* | 2.93 | Neurons | NS | - |  |
| **164** | ***Eps8*** | **2.90** | **Pericytes** | **Up** | **1.81066E-06** |  |
| 165 | *Il13ra1* | 2.89 | Myeloid | Up | - |  |
| 166 | *Plat* | 2.88 | Endothelial cells | Up | 3.14814E-05 |  |
| **167** | ***Aspn*** | **2.87** | **Pericytes** | **Up** | **7.93E-09** |  |
| 168 | *Fam107b* | 2.87 | Oligodendrocytes | NS | - |  |
| 169 | *Mmp14* | 2.87 | Endothelial cells | Up | 2.03E-12 |  |
| 170 | *Axl* | 2.86 | Myeloid | Up | - |  |
| 171 | *Fads3* | 2.86 | Astrocytes | NS | - |  |
| 172 | *Gstt1^f^* | 2.84 | Astrocytes | - | - |  |
| 173 | *Btd* | 2.82 | Astrocytes | Up | - |  |
| 174 | *Casq2* | 2.80 | Neurons | NS | - |  |
| 175 | *4933426M11Rik^g^* | 2.78 | Myeloid | Up | - |  |
| **176** | ***Phldb2*** | **2.78** | **Pericytes** | **NS** | **7.08E-09** |  |
| 177 | *Cd63* | 2.75 | Neoplastic cells | Up | - |  |
| 178 | *Slc25a33* | 2.72 | Astrocytes | NS | - |  |
| 179 | *Gucy1a3* | 2.68 | Neurons | NS | - |  |
| **180** | ***Cyth3*** | **2.66** | **Pericytes** | **NS** | **1.44813E-06** |  |
| 181 | *Slc7a2* | 2.66 | Astrocytes | NS | - |  |
| 182 | *Lims1* | 2.66 | Myeloid | Up | - |  |
| 183 | *M6pr* | 2.64 | Neurons | Up | - |  |
| 184 | *Ugp2* | 2.64 | Astrocytes | NS | - |  |
| 185 | *Marcks* | 2.63 | OPCs | Up | - |  |
| 186 | *Arhgef25* | 2.62 | Neoplastic cells | Down | - |  |
| 187 | *Adk* | 2.60 | Neurons | NS | - |  |
| 188 | *Asph* | 2.58 | Astrocytes | NS | - |  |
| 189 | *Laptm4a* | 2.56 | Astrocytes | Up | 1.43E-13 |  |
| 190 | *Hlf* | 2.55 | Neurons | Down | - |  |
| **191** | ***Lamc3*** | **2.54** | **Pericytes** | **Up** | **6.00E-09** |  |
| 192 | *Gns* | 2.53 | Myeloid | Up | - |  |
| 193 | *Rasgrp2* | 2.52 | Neurons | NS | - |  |
| 194 | *Itpr1* | 2.50 | Neurons | Down | - |  |
| 195 | *Map1lc3a* | 2.50 | Neurons | Down | - |  |
| **196** | ***Mrc2*** | **2.49** | **Pericytes** | **Up** | **1.86E-13** |  |
| **197** | ***Tenc1*** | **2.48** | **Pericytes** | **Up** | **1.86E-08** |  |
| **198** | ***Pear1*** | **2.46** | **Pericytes** | **NS** | **8.16E-10** |  |
| 199 | *Gatm* | 2.45 | Astrocytes | Up | - |  |
| 200 | *Rgl1* | 2.44 | Astrocytes | NS | - |  |
