# Supplementary material for: Upregulated functional gene expression programmes in tumour pericytes mark progression in patients with low‐grade glioma
Source: Mol Oncol. 2021 Jun 5;16(2):405–21. doi: 10.1002/1878-0261.13016 (PMC8763650; doi:10.1002/1878-0261.13016)
Supplement: Supplementary file 7 — Table S1. PEGs. Table S2. Overlapping correlating genes of the PEGs. [file MOL2-16-405-s004.zip › mol2_13016_Supplementary Table 2 - Overlapping correlating genes.docx]

| **Supplementary Table 2. Overlapping correlating genes of the PEG signatures** | | | |  |  |  |
| --- | --- | --- | --- | --- | --- | --- |
| Gene lists containing overlapping correlating genes specific for the PEG signatures. | | | | | |  |
|  | **Upregulated PEGs** | |  |  | **Non-significant PEGs** | |
| Vascular | Immune PC | Mϕ-PEG |  | Vascular (1) | Vascular (2) | Immune PC |
| ACVRL1 | EVC | RALB |  | LAMC3 | HYAL2 | ROR2 |
| TBXA2R | SLC35D1 | F13A1 |  | BCL6B | TMEM255B | NABP1 |
| EXOC3L2 | EPHB4 | FPR2 |  | ACVRL1 | TM4SF1 | ARHGAP24 |
| GPR4 | PKD2 | CCL26 | Vascular (1) | DIPK2B | CYTOR | EMBP1 |
| FLT1 | ADAM9 | PARVB | LAMC3 | LRRC32 | CDC42EP5 | IRF4 |
| TNFAIP8L1 | IQGAP1 | TRIM38 | BCL6B | FOXF2 | KANK3 | CXCL12 |
| TMEM204 | EFNB2 | TPM3 | ACVRL1 | NOX4 | CETP | COL14A1 |
| PLVAP | WWTR1 | NFKBIZ | DIPK2B | ESAM | RAMP2 | HACD4 |
| SMTN | FKBP9 | CR1 | LRRC32 | ENPEP | ADAMTSL3 | TSPAN2 |
| AVPR1A | CHPF2 | LYVE1 | FOXF2 | GRAP | DYSF | ARHGAP28 |
| FZD4 | PTPN12 | CLEC10A | NOX4 | SLC38A11 | TBX2 | DPT |
| GIPC3 | H6PD | FHL2 | ESAM | TMEM204 | INSR | TBC1D9 |
| PDGFB | GALNT2 | CCL20 | ENPEP | ADGRL4 | CYTH3 | PHLDB2 |
| TMEM30B | PTPN9 | IFNGR2 | GRAP | MYCT1 | C1QTNF6 | PSTPIP2 |
| OR2A9P | MYO1E | PRG4 | SLC38A11 | CLEC14A | EBF2 | CMTM7 |
| DCLK3 | HS3ST3B1 | CXCL1 | TMEM204 | GPR4 | SP6 | MME |
| UNC5B | CEMIP | CXCL6 | ADGRL4 | PEAR1 | SERPINH1 | OLFML2B |
| DYSF | ARSJ | FKBP1A | MYCT1 | MCAM | SPON2 | DSE |
| SOX17 | PCSK5 | EGFL6 | CLEC14A | DLL4 | RFLNB | PTGER4 |
| RFLNB | PLS3 | AHR | GPR4 | PCDH12 | HSD11B2 | RUNX2 |
| CYYR1 | DNAJC3 | SOCS3 | PEAR1 | CDH5 | BARHL1 | SCUBE3 |
| IL3RA | FZD7 | LGALS1 | MCAM | FOXS1 | GRM8 | GATA6 |
| FCMR | POGLUT3 | IL6 | DLL4 | ROBO4 | L1TD1 | TENT5C |
| HECW2 | ZNF217 | TMEM87B | PCDH12 | ARHGEF15 | FOXL2NB | TPBG |
| LINC00924 | SNTB2 | RAB11FIP1 | CDH5 | GIPC3 | AFAP1L1 | COLEC12 |
| CAVIN3 | PYGL | CCR2 | FOXS1 | FLT4 | ANO2 | ARHGEF5 |
| CD40 | RIPK1 | RNF144B | ROBO4 | SCARF1 | LRRC70 | ADAMTS12 |
| ACTA2 | LOC100129034 | TNFAIP8 | ARHGEF15 | MMRN2 | HTRA3 | SULF1 |
| KCNQ1 | ZYX | LY96 | GIPC3 | PLXDC1 | IGFBP7 | ADAMTSL1 |
| TNFRSF4 | TRIP12 | SMIM3 | FLT4 | PECAM1 | MPZL2 | FBLN1 |
| NODAL | C16ORF72 |  | SCARF1 | RGS5 | TES | BMP4 |
| GALNT18 | MCL1 |  | MMRN2 | NR5A2 | LRRC36 | MYLIP |
| ST3GAL1 | VASN |  | PLXDC1 | EGFL7 | RERGL | PRICKLE1 |
| SP6 | CUL7 |  | PECAM1 | NPR1 | SPATA22 | STK26 |
| NPR1 | NFKB1 |  | RGS5 | CCDC3 | UQCR11 | TMEM200A |
| CYSLTR2 | NFATC4 |  | NR5A2 | ITGA1 | OVCA2 | VIT |
| C2CD4B | BCL3 |  | EGFL7 | ANGPT2 | NDUFA13 | CD8A |
| IGF2R | PDIA4 |  | NPR1 | DCLK3 | CA5A | WNT5A |
| PLEK2 | DPYD |  | CCDC3 | CD248 | MYL12B | CCL4 |
| PFN1 | SLC2A10 |  | ITGA1 | COL4A1 | ARPC4 | FOLR2 |
| MLKL | PAM |  | ANGPT2 | VWF | SFTA1P | EPB41L3 |
| CDA | GPX8 |  | DCLK3 | PDGFRB | FAM3D | PKP2 |
| TRPV2 | CALU |  | CD248 | NOTCH4 | NDUFA3 | SRL |
| VWA1 | SLC30A5 |  |  | RASIP1 | EIF3K | GCSAM |
| FHL5 | SIX5 |  |  | TIE1 | BORCS8 | BMF |
| GUCY1B1 | FBLN7 |  |  | SH2D3C | AKAP2 | RASGEF1B |
| SIPA1 | LAMB2 |  |  | ACE | TSPAN9 | SGCD |
| GIMAP8 | SWAP70 |  |  | FOXL1 | STRA6 | CD55 |
| CRISPLD2 | OSMR |  |  | FAM241A | PCOLCE | MSX2 |
| MED15 | CCDC102A |  |  | FAM162B | HPGD | CYP7B1 |
| PPM1F | VIM |  |  | ERICH4 | CAVIN3 | HTR2B |
| PRND | GALNT4 |  |  | ECSCR | EGFLAM | POPDC3 |
| SIX2 | RIN1 |  |  | TM4SF18 | PPM1H | CCL3 |
| ADAMTS4 | NOD1 |  |  | CD34 | SEMA3G | Mar1 |
| SEMA7A | EFEMP2 |  |  | FOXC2 | KSR1 | PTGER2 |
| TMC4 | FZD1 |  |  | HMCN1 | ZNF618 | TNFAIP8 |
| VASH1 |  |  |  | OR2A9P | DOCK9 | GPC3 |
| GUCY1A2 |  |  |  | MYO1B | GDPD5 | SPOPL |
| MICALL1 |  |  |  | CCM2L | GPER1 | ABI3BP |
| MEF2D |  |  |  | SOX18 | C2CD4B | SLC7A8 |
| ADAMTSL3 |  |  |  | TMEM74B | LZTS1 | FOXP1 |
| JUP |  |  |  | PROSER2 | ST3GAL2 | SLC16A5 |
| INSR |  |  |  | FLT1 | PTP4A3 | MAFB |
| ANKRD52 |  |  |  | TNFAIP8L1 | PCDH1 | C16ORF54 |
| SLC35E1 |  |  |  | N4BP3 | CHFR | AIF1 |
| GATAD2A |  |  |  | GJA4 | RNASE10 | PLXDC2 |
|  |  |  |  | MAGEA12 | FKBP1A | SYK |
|  |  |  |  | NDUFA4L2 | GALNT18 | IL16 |
|  |  |  |  | TRPC6 | TMEM117 | KCNK6 |
|  |  |  |  | MANCR | POSTN | MAP3K20 |
|  |  |  |  | IL3RA | PHLDA2 | GXYLT2 |
|  |  |  |  | HTR1F | STC1 | CR1 |
|  |  |  |  | TMEM30B | PPEF1 | F13A1 |
|  |  |  |  | ITGA10 | DCN | GIPC2 |
|  |  |  |  | FZD4 | ASB9 | IL1R1 |
|  |  |  |  | PRR16 | TCN2 | ANTXR2 |
|  |  |  |  | TNFRSF4 | TAGLN | PLAGL1 |
|  |  |  |  | FAM43A | ARPC2 |  |
|  |  |  |  | SEMA3F | FCN3 |  |
|  |  |  |  | NOS3 | MRPL52 |  |
|  |  |  |  | HSPG2 | BRK1 |  |
|  |  |  |  | HECW2 | COX7A2 |  |
|  |  |  |  | KDR | MRPS28 |  |
|  |  |  |  | CYYR1 | CGRRF1 |  |
|  |  |  |  | DPEP1 | SDHAF1 |  |
|  |  |  |  | COX4I2 | RAB4B |  |
|  |  |  |  | TNFRSF10D | WDR61 |  |
|  |  |  |  | AVPR1A | NDUFB7 |  |
|  |  |  |  | ANO1 | ATP5MPL |  |
|  |  |  |  | HIGD1B | TBCB |  |
|  |  |  |  | CSAG1 | NDUFB1 |  |
|  |  |  |  | NODAL | NDUFA11 |  |
|  |  |  |  | JAG2 | COMMD7 |  |
|  |  |  |  | HIC1 | UQCRFS1 |  |
|  |  |  |  | RNF152 | SELENOW |  |
|  |  |  |  | HSPA12B | BABAM1 |  |
|  |  |  |  | ADAMTSL2 | MCRIP1 |  |
|  |  |  |  | PXDNL | COX6B1 |  |
|  |  |  |  | COL4A2 | FDX2 |  |
|  |  |  |  | UNC5B | COX7A1 |  |
|  |  |  |  | EXOC3L2 | NEDD8 |  |
|  |  |  |  | COL18A1 | LYRM4 |  |
|  |  |  |  | ETS1 | COX4I1 |  |
|  |  |  |  | SPAAR | TMA7 |  |
|  |  |  |  | LXN | SLC38A5 |  |
|  |  |  |  | MMRN1 | RGCC |  |
|  |  |  |  | NOSTRIN | RUNDC3B |  |
|  |  |  |  | EMCN | PRSS3 |  |
|  |  |  |  | KRT18 | ABCG2 |  |
|  |  |  |  | LINC01139 | TMEM45B |  |
|  |  |  |  | FUT1 | ITM2A |  |
|  |  |  |  | FOXQ1 | MYL6 |  |
|  |  |  |  | TP53I11 | ATP5MC3 |  |
|  |  |  |  | JUP | SLC9A1 |  |
|  |  |  |  | F2RL3 | EPAS1 |  |
|  |  |  |  | NMUR1 | FOXC1 |  |
|  |  |  |  | BGN | ADAM12 |  |
|  |  |  |  | COL5A3 | PALM2-AKAP2 |  |
|  |  |  |  | PXDN | MSMP |  |
|  |  |  |  | LAMA4 | C20ORF202 |  |
|  |  |  |  | OLFML2A | NAALADL1 |  |
|  |  |  |  | CARMN | KCNE3 |  |
|  |  |  |  | OR51E1 | PSMD8 |  |
|  |  |  |  | LINC00924 | C14ORF119 |  |
|  |  |  |  | FHL5 | FAM110D |  |
|  |  |  |  | SPINK8 | CRIP1 |  |
|  |  |  |  | CDA | TMEM37 |  |
|  |  |  |  | CLEC1A | ISOC2 |  |
|  |  |  |  | USHBP1 | RAB5C |  |
|  |  |  |  | MYLK2 | OAZ1 |  |
|  |  |  |  | ICAM2 | BAX |  |
|  |  |  |  | SRARP | MAP11 |  |
|  |  |  |  | CLDN5 | SLC12A9 |  |
|  |  |  |  | TESC | SIDT2 |  |
|  |  |  |  | FBLIM1 | PTH1R |  |
|  |  |  |  | VASH1 | VWA2 |  |
|  |  |  |  | DOCK6 | RASGRP3 |  |
|  |  |  |  | CARD10 | MFSD2A |  |
|  |  |  |  | AGRN | SLC52A3 |  |
|  |  |  |  | RASD2 | MMP28 |  |
|  |  |  |  | FCMR | SLC7A6 |  |
|  |  |  |  | ADAMTS7 | ZNF335 |  |
|  |  |  |  | PLEKHG2 | NBEAL2 |  |
|  |  |  |  | LAMB1 | ADCY6 |  |
|  |  |  |  | ITGA8 | SHANK3 |  |
|  |  |  |  | PLVAP | TBX18 |  |
|  |  |  |  | ESM1 | VSIG2 |  |
|  |  |  |  | MMP9 | SOX7 |  |
|  |  |  |  | PGF | ADAMTS10 |  |
|  |  |  |  | LMOD3 | TEK |  |
|  |  |  |  | HRC | PDE7B |  |
|  |  |  |  | PRND | PTPRB |  |
|  |  |  |  | P2RY14 | OLFML1 |  |
|  |  |  |  | MIR4435-2HG | VASP |  |
|  |  |  |  | PLAC9 | MYL9 |  |
